# Dual Small-Molecule Targeting of SMAD Signaling Stimulates Human Induced Pluripotent Stem Cells toward Neural Lineages

**DOI:** 10.1371/journal.pone.0106952

**Published:** 2014-09-10

**Authors:** Methichit Wattanapanitch, Nuttha Klincumhom, Porntip Potirat, Rattaya Amornpisutt, Chanchao Lorthongpanich, Yaowalak U-pratya, Chuti Laowtammathron, Pakpoom Kheolamai, Niphon Poungvarin, Surapol Issaragrisil

**Affiliations:** 1 Siriraj Center of Excellence for Stem Cell Research, Faculty of Medicine Siriraj Hospital, Mahidol University, Bangkok, Thailand; 2 Division of Hematology, Department of Medicine, Faculty of Medicine Siriraj Hospital, Mahidol University, Bangkok, Thailand; 3 Division of Cell Biology, Department of Pre-clinical Sciences, Faculty of Medicine, Thammasat University, Pathumthani, Thailand; 4 Division of Neurology, Department of Medicine, Faculty of Medicine Siriraj Hospital, Mahidol University, Bangkok, Thailand; Instituto Butantan, Brazil

## Abstract

Incurable neurological disorders such as Parkinson’s disease (PD), Huntington’s disease (HD), and Alzheimer’s disease (AD) are very common and can be life-threatening because of their progressive disease symptoms with limited treatment options. To provide an alternative renewable cell source for cell-based transplantation and as study models for neurological diseases, we generated induced pluripotent stem cells (iPSCs) from human dermal fibroblasts (HDFs) and then differentiated them into neural progenitor cells (NPCs) and mature neurons by dual SMAD signaling inhibitors. Reprogramming efficiency was improved by supplementing the histone deacethylase inhibitor, valproic acid (VPA), and inhibitor of p160-Rho associated coiled-coil kinase (ROCK), Y-27632, after retroviral transduction. We obtained a number of iPS colonies that shared similar characteristics with human embryonic stem cells in terms of their morphology, cell surface antigens, pluripotency-associated gene and protein expressions as well as their *in* *vitro* and *in* *vivo* differentiation potentials. After treatment with Noggin and SB431542, inhibitors of the SMAD signaling pathway, HDF-iPSCs demonstrated rapid and efficient differentiation into neural lineages. Six days after neural induction, neuroepithelial cells (NEPCs) were observed in the adherent monolayer culture, which had the ability to differentiate further into NPCs and neurons, as characterized by their morphology and the expression of neuron-specific transcripts and proteins. We propose that our study may be applied to generate neurological disease patient-specific iPSCs allowing better understanding of disease pathogenesis and drug sensitivity assays.

## Introduction

Neurological disorders including Parkinson’s disease (PD), Huntington’s disease (HD) and Alzheimer’s disease (AD) are considered to be irreversible and incurable due to progressive neural loss and dysfunction [1]. Although neural stem cells (NSCs) in the brain can be activated to proliferate and differentiate to mature neurons, which can migrate toward the site after neural degeneration or injury, the number of neural cells normally derived from endogenous NSCs appears inadequate for the replacement of neural loss [2]. Various cell sources both from fetal and adult NSCs have been applied to neural transplantation but their limited proliferation capacity *in* *vitro* has hindered clinical applications [3].

Over the last decade, human embryonic stem cells (hESCs) have provided a great promise not only as an unlimited renewable source of surrogate cells to repair damaged tissues, but also as a model to study embryonic development and disease mechanisms. Nevertheless, the derivation of hESCs requires human oocytes and subsequent destruction of human embryos, which raise significant ethical concerns. Recent advances in somatic cell reprogramming have provided unlimited numbers of patient-specific pluripotent stem cells [4], [5]. The induced pluripotent stem cells (iPSCs) are comparable to hESCs in terms of their self-renewal and differentiation potential without the ethical issues and immunological rejection when used for autologous transplantation.

Several attempts have been made to differentiate human pluripotent stem cells (hPSCs) to neural progenitor cells (NPCs), which can differentiate further to all neural subtypes including neurons and glial cells [6]. The common neural differentiation protocol has been demonstrated by the formation of embryoid bodies (EB), which is simple, cost-effective and scalable, but heterogeneous cell populations are also generated within the EBs [7]. Co-culturing with mouse mesenchymal stromal cell lines such as PA6 and MS5 cells has been demonstrated to induce neural differentiation by their secretory factors. However, the clinical application of this method has been impeded by the risk of animal cell contamination and by the fact that the secretory factors at play are undefined [8].

To overcome these limitations, a differentiation strategy using serum-free defined factors is essential [9]. Using the knowledge of factors and signaling pathways involving in fetal neural development, hPSCs can be induced to efficiently differentiate into neural lineages. Several studies in *Xenopus laevis* indicated that the inhibitors of bone morphogenetic protein (BMP) including Noggin, Follistatin and Chordin play an important role during neural development of embryo through the SMAD signaling pathway [10]–[12]. In adult mouse brain, Noggin has been demonstrated to be an essential neural-inducing factor and remarkably expressed in nervous system [13]. The addition of recombinant Noggin improved the efficiency of neural conversion of hESCs in culture [14]. Previously, a small molecule, SB431542, has been shown to enhance the neural differentiation of hPSCs through the inhibition of transforming growth factor-beta (TGFβ) pathway, which results in the downstream inhibition of SMAD signaling [15]. The synergistic action of Noggin and SB431542 has been shown to rapidly drive cell fate alteration from pluripotent to NPC stage, which can be further committed to specific neural cell types such as cortical neurons [16], [17]. This strategy could thus enhance the potential use of iPSC-derived neurons in future clinical applications.

The present study aims to establish a protocol for iPSC generation and differentiation to NPCs and mature neurons through dual-action of small molecules during neuronal induction period. This rapid and efficient differentiation strategy could be further used for the generation of patient-specific iPSC lines from patients’ fibroblasts with several neurological diseases and would provide an alternative source of pluripotent stem cells for the study of molecular mechanisms, early embryonic developmental pathways [18], the pathological basis of genetic disorders as well as toxicology or pharmacology testing upon neuronal lineage differentiation in future studies [19], [20].

## Materials and Methods

### Cell culture

Human dermal fibroblasts (HDFs) (ScienCell, USA) and human foreskin fibroblasts (HFFs) (ATCC) were maintained in fibroblast medium: DMEM supplemented with 10% fetal bovine serum (FBS) (Lonza, Switzerland), 1x GlutaMAX and 25 U/ml penicillin, 25 mg/ml streptomycin. The hESC line (Chula2.hES) [21] and iPSCs were maintained on γ-irradiated (45 Gy) HFF (iHFF) and cultured in hESC medium, which contains knockout Dulbecco’s modified Eagle’s medium (KO-DMEM)-high glucose, 20% knockout serum replacer, 2 mM L-glutamine, 0.1 mM non-essential amino acids, 0.1 mM 2-mercaptoethanol, 1x insulin-transferrin-selenium, 25 U/ml penicillin, 25 mg/ml streptomycin and 4 ng/ml basic fibroblast growth factor (bFGF). hESCs and iPSCs were subcultured approximately once every 5 days by incubation with 0.05% Trypsin for 2 min as described previously [22] (all reagents were from Invitrogen, USA). Alternatively, hESCs and iPSCs were maintained in mTESR1 medium (Stem Cell Technologies, Canada) on Matrigel (BD Bioscience, USA)-coated cell culture plates. hESCs and iPSCs were subcultured approximately once every 5 days by incubation with 1 mg/ml Dispase (Stem Cell Technologies, Canada) for 7 min as per standard hESC procedures.

### Generation of induced pluripotent stem cells

**Figure 1:**
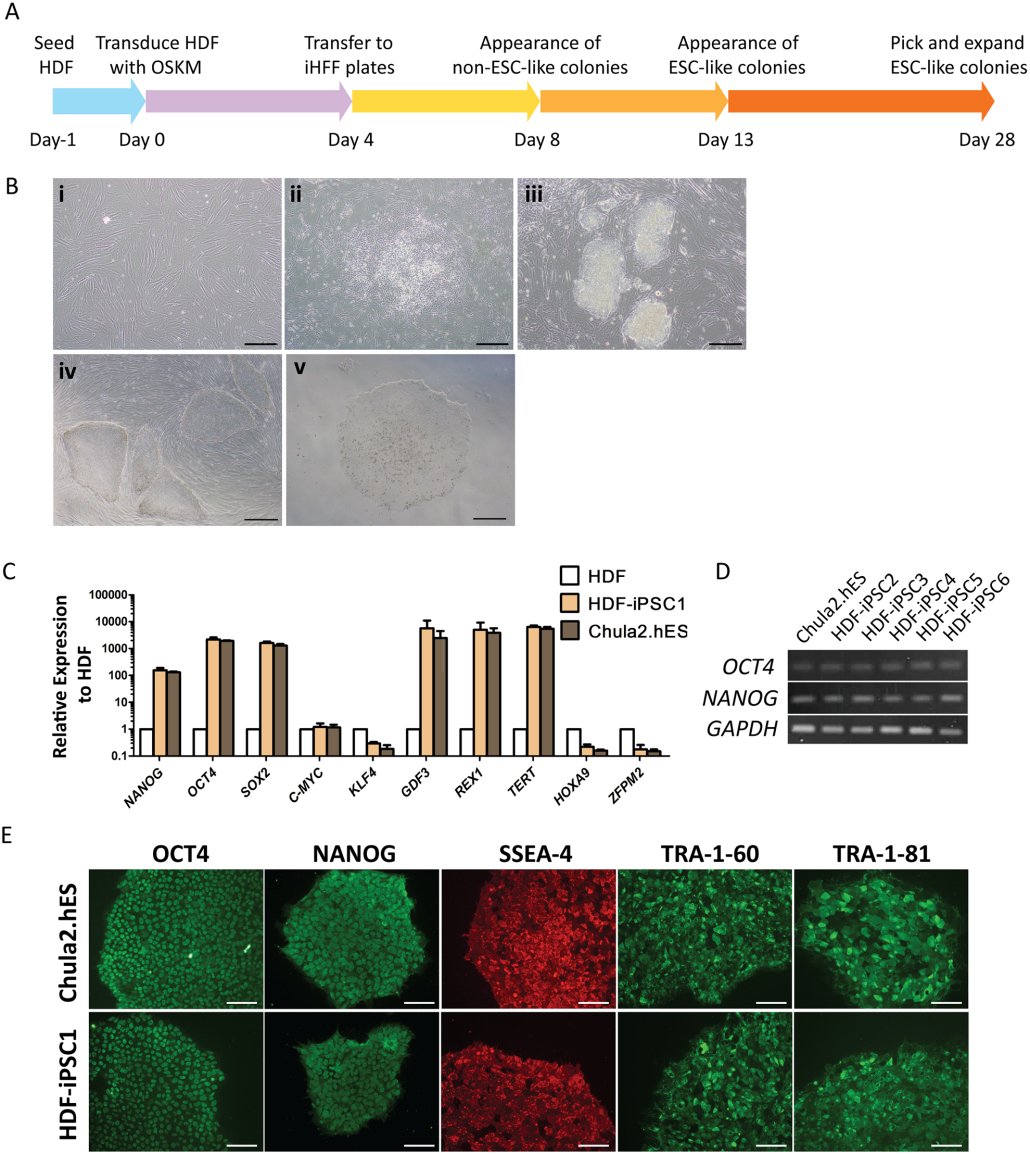
Generation and characterization of iPSCs from human dermal fibroblasts (HDFs). (A) Schematic diagram represents a protocol to generate iPSCs from HDFs by retroviral transduction. (B) Morphology of HDFs on day 1 post-transduction (i), non ESC-like colonies on day 8 (ii), ESC-like colonies on day 13 (iii), iPS colonies on iHFF plate (iv) and an iPS colony on Matrigel-coated plate (v), scale bar (i) and (iii) = 200 µm, (ii), (iv) and (v) = 500 µm. (C) RT-qPCR analysis of pluripotency- and fibroblast-associated genes in HDF, HDF-iPSC1 and Chula2.hES cells. (D) RT-PCR analysis of pluripotent genes, OCT4 and NANOG, of all the established iPS clones, HDF-iPSC2-6, as compared to Chula2.hES cells. (E) Representative immunofluorescent images of pluripotent transcription factors and cell surface antigens of HDF-iPSC1 as compared to Chula2.hES cells, scale bar = 100 µm.

Moloney-based retroviral vectors (pMXs) containing human complementary DNAs (cDNAs) of OCT4, SOX2, KLF4 and C-MYC (Cell Biolabs, USA) were transfected into a retroviral packaging cell line, Platinum-A cells (Cell Biolabs) using FuGENE HD transfection reagent (Promega Corporation, USA). One day before infection, passages 6–8 of the HDF were seeded at 100,000 cells per well of 6-well plate. The viral supernatant from each plate was collected 48 h post-transfection and the plates were replaced with fresh medium for consecutive infections. The viral supernatant was filtered through a 0.45 µm pore-size filter and supplemented with 4 µg/ml polybrene before infecting HDFs. For experimental control, we transfected Platinum-A cells with pMX-GFP retroviral vector (Cell Biolabs) and transduced HDFs with the viral supernatant. The cells were infected 3 times at 12 h intervals. After infection, 0.5 mM valproic acid (VPA) (Merck, USA) was supplemented to the culture for the first 10 days to optimize the colony formation efficiency [23]. On day 4 post-infection, these cells were treated with 10 µM Y-27632 for an hour prior to trypsinization and transferred onto iHFF in hESC medium supplemented with 0.5 mM VPA and 10 µM Y-27632 (Merck, USA). The cells were cultured until ES-like colonies appear. These colonies were picked and transferred to a fresh iHFF feeder plate for expansion (Figure 1A). The iPSC lines were named HDF-iPSCs and adapted to feeder-free system for further expansion and characterization.

### *In vitro* differentiation of human iPSCs

Spontaneous differentiation via embryoid body (EB) formation was performed by treating HDF-iPSCs with 1 mg/ml Dispase for 30 min until colonies lifted off the plate. The colonies were washed in KO-DMEM basal medium and transferred to low attachment dish in hESC medium without bFGF supplementation for a week. The EBs were then seeded onto 0.1% gelatin-coated dishes and cultured in the same medium for another 3 weeks. Lineage marker gene and protein expression were examined by quantitative reverse transcription polymerase chain reaction (RT-qPCR) and immunofluorescent staining, respectively.

### *In vitro* neural differentiation of human iPSCs

HDF-iPSCs were induced to differentiate into neuronal lineages as adherent monolayer culture system as previously described with modifications [24]. Briefly, HDF-iPSCs were dissociated into single cells with Accutase. The cells were seeded at a density of 3.5–5×10^4^ cells/cm^2^ in mTESR1 medium on Matrigel-coated plates in order to obtain 100% confluency within one day after seeding. Thereafter, the medium was changed to neural induction medium, which contains a 1∶1 mixture of DMEM/F12 glutamax and neurobasal medium, 1x N2 supplement, 1x B27 supplement, 5 µg/ml insulin, 1 mM L-glutamine, 0.1 mM non-essential amino acids, 0.1 mM 2-mercaptoethanol, 25 U/ml penicillin, 25 mg/ml streptomycin, and supplemented with 200 ng/ml Noggin (R&D system, USA) and 10 µM SB431542 (Tocris Bioscience, USA). The medium was changed every day for 6 days. The neuroepithelial cells (NEPCs) were dissociated with 1 mg/ml Dispase for 3 min and triturated into small clumps. The cells were replated onto laminin-coated plates in neural induction medium. On day 7 of differentiation, the NEPCs were cultured in the neural induction medium minus Noggin and SB431542. The medium was supplemented with 20 ng/ml bFGF for 3–4 days for neural rosette formation. Mature neuronal differentiation was further performed by bFGF removal and cultured on polyornithine and laminin-coated plates. The medium was changed every 2–3 days.

### Gene expression analysis by RT-qPCR

Total RNA was prepared using TRIzol reagent (Invitrogen Corporation). cDNA was prepared using 2 µg of RNA and reverse transcribed using SuperScript III First-Strand Synthesis System and Oligo (dT) primers (Invitrogen Corporation). qPCR analysis was carried out on 7500 Fast Real-time PCR machine (Applied Biosystems, California) using Power SYBR Green PCR Master Mix (Applied Biosystems). The PCR reaction consisted of 1x SYBR Green PCR master mix, 200 nM of forward and reverse primers, 1 µl of template cDNA. The total volume was adjusted to 20 µl with nuclease-free water. Initial enzyme activation was performed at 95°C for 10 min, followed by 40 cycles of denaturation at 95°C for 15 s, and primer annealing/extension at 60°C for 1 min. Melting curve analysis was performed to determine primer specificity. The relative expression of each gene was normalized against the house-keeping gene product, Glyceraldehyde 3-phosphate dehydrogenase (GAPDH). Primer sequences are provided in Table S1 (Supplementary data). Each experiment was carried out 3 times.

### Immunofluorescent staining

Cells were washed with phosphate buffered saline (PBS) and fixed with 4% paraformaldehyde for 20 min. After washing with PBS, cells were permeabilized with 0.1% Triton X-100 (Sigma) in PBS for 15 min for intracellular staining. Cells were then blocked with 3% bovine serum albumin (BSA) (Sigma) in PBS for 1 h at room temperature and incubated with primary antibodies diluted in 1% (w/v) BSA in PBS overnight at 4°C. Cells were washed with PBS three times and stained with secondary antibodies for 1 h at room temperature in the dark. The following primary and secondary antibodies were used: mouse anti-human OCT4 (AbD Serotec, UK), rabbit anti-human NANOG (Millipore), Alexa Fluor 488 anti-human TRA-1-60 antibody (BioLegend), mouse anti-human TRA-1-81 (Millipore), mouse anti-human Stage-Specific Embryonic Antigen-4 (SSEA-4) (Millipore), mouse anti-human NESTIN (Millipore), rabbit anti-human TUJ1 (Covance), mouse anti-human α-fetoprotein (Calbiochem), mouse anti-human actin (muscle) clone HHF35 (AbD Serotec), mouse anti-human PAX6 (Millipore), Alexa Fluor 488 γoat anti-mouse IgG (H+L), Alexa Fluor 488 goat anti-rabbit IgG (H+L) and Alexa Fluor 568 goat anti-mouse IgG (H+L) (Life Technologies). Nuclei were stained with prolong gold DAPI with antifade (Invitrogen). Cells were visualized with an Eclipse TE2000 fluorescent microscope (Nikon, Japan).

### Teratoma formation

All animal experiments were performed with an approval of Siriraj Animal Care and Use Committee (SiACUC), number 002/2556. Briefly, HDF-iPSCs were treated with 10 µM of Y-27632 for 1 h prior to harvesting with accutase. Cells were resuspended at 1×10^7^ cells/200 µl of cold 30% (v/v) Matrigel in KO-DMEM basal medium and implanted intramuscularly into 6–8 weeks old nude (*Foxn1nu*) mice obtained from National Laboratory Animal Center, Mahidol University. Chula2.hES cells were also injected into nude mice as a positive control. Teratomas were removed 8 weeks post-transplantation, fixed in 10% neutral buffered formalin and embedded in paraffin wax. Samples were sectioned and examined by hematoxylin and eosin (H&E) staining.

### Karyotypic analysis

Chromosomal analysis was performed at Siriraj Central Cytogenetic Laboratory, Faculty of Medicine Siriraj Hospital, Mahidol University. Briefly, HDF-iPSCs were cultured in T-25 flask on Matrigel in mTESR1 medium for 5 days until reaching 80% confluency. Cells were treated with colcemid to block the cell division at metaphase stage and harvested for analysis using a standard protocol for G-banded karyotyping.

### Statistical analysis

Data are presented as mean ± SD from three independent experiments. The statistics generated in this study were performed using GraphPad Prism 5 (GraphPad Software, Inc).

## Results

### Generation of human iPSCs from human dermal fibroblasts

We generated human iPSCs using retroviral transduction with 4 vectors encoding human cDNA of OCT4, SOX2, KLF4 and C-MYC into human dermal fibroblasts (Figure 1A). On day 4 post-infection, the infected cells were transferred to iHFF plates. Non ESC-like colonies with cobblestone phenotype appeared as early as day 8 post-infection (Figure 1B-ii). We observed small ESC-like colonies from days 13–28 post-infection (Figure 1B-iii). More than 30 iPS colonies were observed from 2 wells of a 6-well plate in one experiment. Those colonies were physically dissected and transferred to iHFF feeder plates for expansion. After 3 passages, the cells were adapted to feeder-free condition. The HDF-iPS colonies were compact with clearly defined edges on both iHFF feeders and feeder-free conditions (Figure 1B-iv and v, respectively).

### HDF-iPSCs display typical characteristics of hESCs

We isolated a total of 6 clones (HDF-iPSC1-6); one clone (HDF-iPSC1) was fully characterized. The undifferentiated HDF-iPSC1 cell line typically expressed endogenous pluripotency-associated genes including *NANOG, OCT4, SOX2, GDF3, REX1* and *TERT* at similar levels to those of Chula2.hES cells as shown by RT-qPCR. These markers were markedly upregulated as compared to those of the parental HDF. Expression of fibroblast-specific markers including *HOXA9* and *ZFPM2* were downregulated as compared to those of the HDF (Figure 1C). Other iPS clones, HDF-iPSC2-6, also expressed *OCT4* and *NANOG* as determined by standard RT-PCR (Figure 1D). Moreover, the HDF-iPSC1 cells also expressed transcription factors, OCT4 and NANOG, and specific cell surface antigens, SSEA-4, TRA-1-60 and TRA-1-81, with a similar pattern to those observed in Chula2.hES cells, as demonstrated by immunofluorescent staining (Figure 1E).

**Figure 2:**
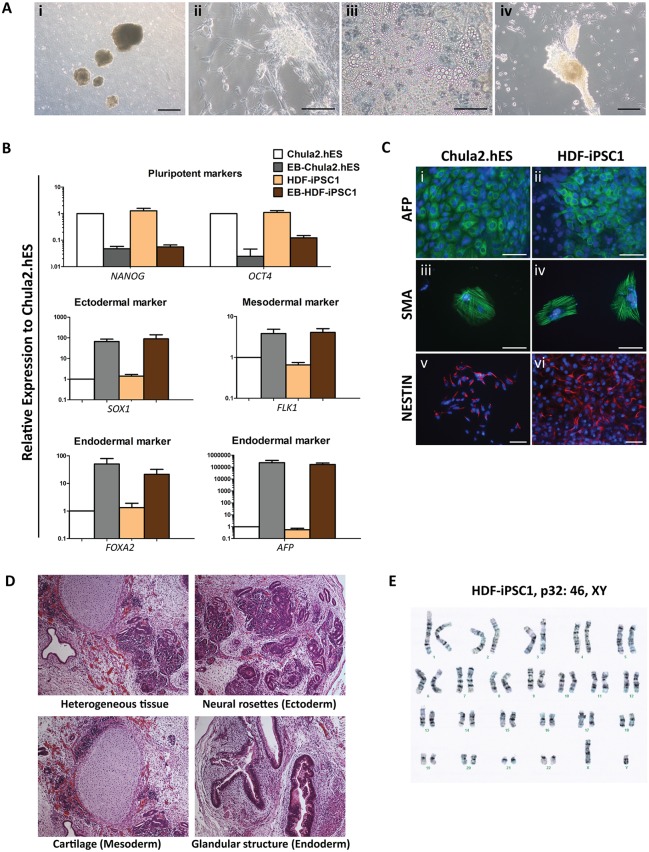
*In vitro* and *in* *vivo* differentiation, and karyotypic analysis of HDF-iPSC1 cells. (A) Representative micrographs show morphology of the embryoid body (EB) of HDF-iPSC1 cells on day 1 in suspension culture (i), differentiated EB turned into neurons (ii), adipocytes (iii) and beating cardiomyocytes (iv), scale bar (i) = 500 µm, (ii) and (iii) = 50 µm and (iv) = 200 µm. (B) RT-qPCR analysis of pluripotent and lineage markers of the differentiated EB generated from HDF-iPSC1 and Chula2.hES cells as compared to the undifferentiated HDF-iPSC1 and Chula2.hES cells. (C) Immunofluorescent analysis showed the expression of lineage markers, AFP (endoderm), SMA (mesoderm) and NESTIN (ectoderm) of the EB outgrowth generated from HDF-iPSC1 cells after 4 weeks of differentiation, scale bar (i–iv) = 50 µm, (v) and (vi) = 100 µm. (D) Hematoxylin and eosin (H&E) staining of teratomas derived from HDF-iPSC1 cells at 8 weeks post-implantation into nude mice. The teratomas contained heterogeneous tissues of all three embryonic germ layers including neural rosettes (ectoderm), cartilage (mesoderm) and glandular structure (endoderm). (E) Representative karyotype analysis of HDF-iPSC1 cells at passage 32 shows normal karyotype (46, XY).

The *in* *vitro* pluripotency of HDF-iPSC1 cells was assessed by spontaneous differentiation via embryoid body (EB) formation. The HDF-iPSC1 cells formed EBs in suspension culture in a similar manner to hESCs. The EBs were then seeded onto gelatin-coated plate where the cells were seen to differentiate to neurons, adipocytes and beating cardiomyocytes (Figure 2A and Movie S1). Expression of pluripotency marker genes, *NANOG* and *OCT4*, were downregulated upon differentiation whereas the lineage-specific marker genes, *SOX1* (ectoderm), *FLK1* (mesoderm), *FOXA2* and *AFP* (endoderm) were upregulated after 4 weeks of differentiation, when compared to undifferentiated HDF-iPSC1 or Chula2.hES cells (Figure 2B). The differentiated HDF-iPSC1 cells also expressed markers of three embryonic germ layers including AFP (endoderm), SMA (mesoderm) and NESTIN (ectoderm) as shown by immunofluorescent staining (Figure 2C).

We then examined the pluripotency of HDF-iPSC1 cells *in* *vivo* by injecting 1×10^7^ cells intramuscularly into nude mice. Histological analysis at 8 weeks post-transplantation revealed that the HDF-iPSC1-derived teratomas consisted of tissues representing all 3 embryonic germ layers including neural rosettes/epithelium (ectoderm), cartilage tissue (mesoderm) and glandular tissue (endoderm) (Figure 2D). Karyotyping of cultured HDF-iPSC1 cells at passage 32 was analyzed by chromosomal counting and G-banded karyotyping. The results from 25 metaphase-stage cells indicated that the HDF-iPSC1 cells were karyotypically normal (46, XY) (Figure 2E).

### *In vitro* differentiation of HDF-iPSC1 to neural lineages

**Figure 3:**
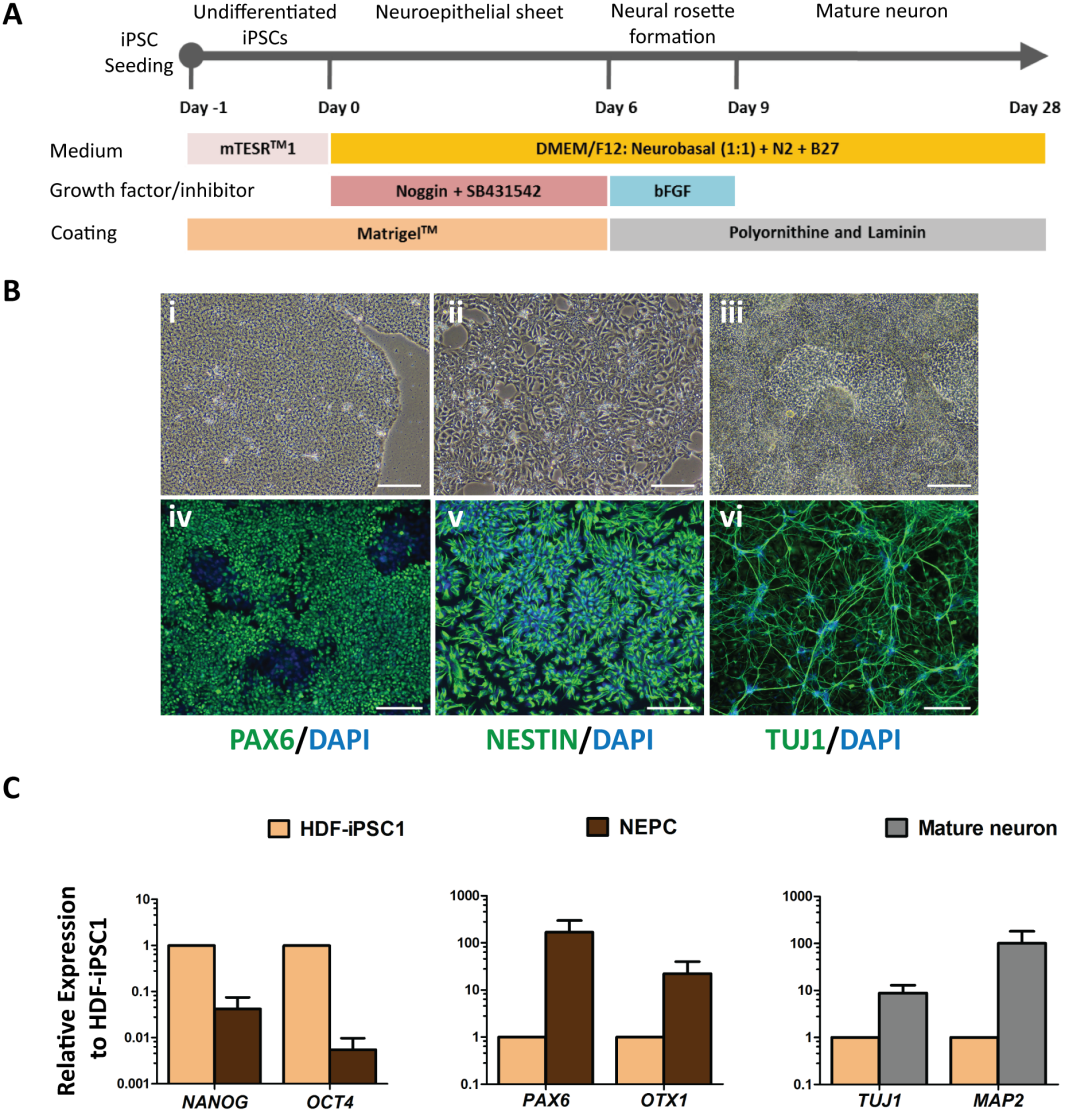
Neural differentiation of HDF-derived iPSCs and characterization of differentiated neurons. (A) Schematic diagram represents the strategy to differentiate HDF-derived iPSCs into neural lineages using SMAD signaling inhibitors. (B) Morphological changes in HDF-iPSC1 cultures during neural differentiation; undifferentiated HDF-iPSC1 cells on day -1 (i), differentiated cells on day 2 (ii) and day 6 (iii), representative immunofluorescent images show the NEPC on day 6 (iv), NPC on day 9 (v) and post-mitotic neuron on day 28 (vi) of differentiation as examined by PAX6, NESTIN and TUJ1 markers, respectively. Cell nuclei were stained with DAPI. Scale bar = 100 µm. (C) RT-qPCR analysis of pluripotency- and neural-associated genes in NEPCs on day 6 and mature neuron on day 28 of differentiation as compared to those of undifferentiated HDF-iPSC1 cells (arbitrarily set at 1).

We generated NPCs of the cerebral cortex from HDF-iPSC1 cells in monolayer culture using dual SMAD signaling inhibitors (Figure 3A). HDF-iPSC1 cells were induced to differentiate into NEPC stage, which was identified by their morphology showing a tightly packed neuroepithelial sheet with small nuclei (Figure 3B-iii). To select an appropriate timing for NEPC generation, we examined the expression levels of NEPC marker, PAX6, on days 4, 5 and 6 of neural induction by immunofluorescent staining. The number of PAX6-positive cells gradually increased and was highest on day 6 of differentiation (Figure S1 and Figure 3B-iv). This pattern of PAX6 expression correlated with data from RT-qPCR analysis on days 4 and 6 of differentiation, which demonstrated that the expression level of *OCT4* was downregulated and that of *PAX6* was upregulated on day 6 (Figure S2). The expression level of neuroepithelial marker gene, *OTX1*, was also upregulated after 6 days of differentiation when compared with undifferentiated HDF-iPSC1 cells (Figure 3C).

Thereafter, we induced NEPCs to differentiate to NPCs, which is radially organized as a neural rosette structure. These iPSC-derived NPCs were expanded in monolayer culture using bFGF supplementation and expressed the transcription factor, NESTIN, as demonstrated by immunofluorescent staining (Figure 3B-v). After bFGF removal, a majority of NPCs were further differentiated to post-mitotic mature neurons, which exhibited neural outgrowth and formed neural network, as characterized by immunofluorescent staining with TUJ1 (Figure 3B-vi). Likewise, RT-qPCR analysis demonstrated that the expression levels of neural-specific marker genes, *TUJ1* and *MAP2*, were significantly upregulated as compared to those of the undifferentiated HDF-iPSC1 cells (Figure 3C).

## Discussion

The objective of our study was to develop a strategy to generate human iPSC-derived neural cell lineages to serve as an unlimited renewable source for repair of damaged tissues and also as *in* *vitro* models in which to study the underlying mechanisms of neurological diseases. The reprogramming efficiency was enhanced by transducing the HDFs with viral supernatant three times every 12 hours and supplementing the transduced cells with the histone deacetylase inhibitor, VPA, for 10 days post-infection. VPA has been shown to be the most potent chemical for augmenting the reprogramming efficiency of mouse embryonic fibroblasts and human fibroblasts compared with other histone deacetylase inhibitors, such as suberoylanilide hydroxamic acid (SAHA) and trichostatin A (TSA), and DNA methyltransferase inhibitor, 5′-azacytidine [23], [25]. We observed a greater number of iPS colonies in the transduced fibroblasts, which were supplemented with VPA, when compared to those without VPA (data not shown). In addition, before transferring the transduced HDFs to a new iHFF feeder plate, we supplemented the culture with Y-27632, an inhibitor of p160-Rho associated coiled-coil kinase (ROCK), which has been shown to prevent dissociation-induced apoptosis in several studies [22], [26]–[29]. The use of ROCK inhibitor improved iPSC survival and adherence to the feeder layer. The established HDF-iPSCs shared similar characteristics with Chula2.hES line, in terms of their morphology, cell surface antigens, pluripotency-associated gene and protein expressions as well as the *in* *vitro* differentiation potential to generate derivatives of three embryonic germ layers.

This study also demonstrates the neural differentiation of HDF-derived iPSCs on adherent monolayer culture with defined factors. Our optimized culture condition could induce HDF-iPSCs to differentiate to NEPCs, NPCs and mature neurons. By using a combination of Noggin and SB431542, HDF-iPSC1 cells rapidly lost their pluripotency and became committed to a neural cell fate, which can be observed from day 4 after induction. Our result was consistent with the previous study, which demonstrated that dual SMAD inhibition greatly induced loss of *OCT4* expression [16]. In contrast to previous studies where neural induction was performed for 8–12 days prior to neural rosette formation [16], [17], [24], in our system, the differentiated cells cultured beyond 6 days were densely packed and started to detach at the edge of culture plate (data not shown). Therefore, we performed NEPC induction for 6 days and observed the confluent homogenous neuroepithelial sheet of the differentiated cells. These cells progressively lost their *OCT4* expression, strongly expressed *PAX6* and demonstrated their ability to differentiate further into NPCs after NEPC dissociation.

Several studies reported the application of these inhibitors in neural differentiation protocols, either alone or by dual supplementation. The BMP inhibitor, Noggin was previously shown to induce neural differentiation of hESCs under culture conditions using defined factors [30]. By adding Noggin, neurospheres can potentially be generated from hESCs by suppressing the expression of endodermal and mesodermal cell fates in suspension culture [31] while the addition of SB431542, a selective inhibitor of the TGFβ pathway, inhibits SMAD signaling by modulating phosphorylation of ALK4, ALK5 and ALK7 receptors [32]. SB431542 mediated p-Erk(ERK)1/2 signaling that promotes neural conversion, which results in increased NPCs derived from mESCs and hESCs through inhibition of the Activin/ALK4 pathway [33]. Taken together, the combination of Noggin and SB431542 thus endowed a synergistic effect on cell fate alteration from pluripotent stem cells to neural lineages [17]. These dual SMAD inhibitors have been used for several neural differentiation strategies to derive NPCs that can give rise to specific neural cell types such as cortical and dopaminergic neurons [16], [34]. Therefore, these iPSC-derived NPCs may be considered as an unlimited renewable cell source for neurological disease modeling and for cell-based transplantation.

Recently, *in* *vitro* models of neurological diseases have been utilized to uncover the underlying disease mechanisms. Derivation of AD patient-specific iPSCs has provided a model for studying pathogenesis and drug discovery [35]. The isogenic PD patient-derived iPSCs have been successfully generated for modeling genetic and molecular mechanisms of the disease in which the specific pathway was discovered for developing targeted therapy for PD [36]. Moreover, the cytosine adenine guanine (CAG) repeat iPSC lines were established from HD followed by successful targeted gene correction of HD by homologous recombination. The genetically corrected iPSCs gave rise to specific neural subtypes, which reversed disease phenotype of the neural stem cells and could potentially be used for cell replacement therapy in the future [37].

In conclusion, the present study provides a protocol to generate, characterized human iPSC lines from HDF and further differentiate them to mature neurons using dual SMAD signaling inhibitors. The approach described in this study can be used to generate patient-specific PSCs, which can be used to study mechanisms underlying patient’s neurological disease. When combined with an efficient gene correction, these genetically corrected iPSCs can provide an autologous cell source for cell replacement therapy.

## Supporting Information

Figure S1 **Representative immunofluorescent images show PAX6-positive cells on days 4, 5 and 6 of neural induction.**
(TIF)

Figure S2 **RT-qPCR analysis of** ***OCT4*** **and** ***PAX6*** **of differentiated cells on days 4 and 6 of neural induction as compared to those of undifferentiated HDF-iPSC1 cells.**
(TIF)

Table S1 **Primer sequences for RT-qPCR.**
(DOCX)

Movie S1 **Beating cardiomyocytes from differentiated EBs of HDF-iPSC1 cells.**
(M4V)

## References

[1] Hsu YC, Chen SL, Wang DY, Chiu IM (2013) Stem cell-based therapy in neural repair. Biomed J 36: 98–105. 23806879 10.4103/2319-4170.113226

[2] Clarke DL (2003) Neural stem cells. Bone Marrow Transplant 32 Suppl 1S13–17. 12931233 10.1038/sj.bmt.1703937

[3] Jakel RJ, Schneider BL, Svendsen CN (2004) Using human neural stem cells to model neurological disease. Nat Rev Genet 5: 136–144. 14735124 10.1038/nrg1268

[4] Takahashi K, Okita K, Nakagawa M, Yamanaka S (2007) Induction of pluripotent stem cells from fibroblast cultures. Nature protocols 2: 3081–3089. 18079707 10.1038/nprot.2007.418

[5] Takahashi K, Yamanaka S (2006) Induction of Pluripotent Stem Cells from Mouse Embryonic and Adult Fibroblast Cultures by Defined Factors. Cell 126: 663–676. 16904174 10.1016/j.cell.2006.07.024

[6] Zhang SC, Wernig M, Duncan ID, Brustle O, Thomson JA (2001) In vitro differentiation of transplantable neural precursors from human embryonic stem cells. Nat Biotechnol 19: 1129–1133. 11731781 10.1038/nbt1201-1129

[7] Clark AT, Bodnar MS, Fox M, Rodriquez RT, Abeyta MJ, et al (2004) Spontaneous differentiation of germ cells from human embryonic stem cells in vitro. Hum Mol Genet 13: 727–739. 14962983 10.1093/hmg/ddh088

[8] Kawasaki H, Mizuseki K, Nishikawa S, Kaneko S, Kuwana Y, et al (2000) Induction of midbrain dopaminergic neurons from ES cells by stromal cell-derived inducing activity. Neuron 28: 31–40. 11086981 10.1016/s0896-6273(00)00083-0

[9] Ying QL, Nichols J, Chambers I, Smith A (2003) BMP induction of Id proteins suppresses differentiation and sustains embryonic stem cell self-renewal in collaboration with STAT3. Cell 115: 281–292. 14636556 10.1016/s0092-8674(03)00847-x

[10] Smith WC, Harland RM (1992) Expression cloning of noggin, a new dorsalizing factor localized to the Spemann organizer in Xenopus embryos. Cell 70: 829–840. 1339313 10.1016/0092-8674(92)90316-5

[11] Hemmati-Brivanlou A, Kelly OG, Melton DA (1994) Follistatin, an antagonist of activin, is expressed in the Spemann organizer and displays direct neuralizing activity. Cell 77: 283–295. 8168135 10.1016/0092-8674(94)90320-4

[12] Sasai Y, Lu B, Steinbeisser H, Geissert D, Gont LK, et al (1994) Xenopus chordin: a novel dorsalizing factor activated by organizer-specific homeobox genes. Cell 79: 779–790. 8001117 10.1016/0092-8674(94)90068-x PMC3082463

[13] Valenzuela DM, Economides AN, Rojas E, Lamb TM, Nunez L, et al (1995) Identification of mammalian noggin and its expression in the adult nervous system. J Neurosci 15: 6077–6084. 7666191 10.1523/JNEUROSCI.15-09-06077.1995 PMC6577675

[14] Dottori M, Pera MF (2008) Neural differentiation of human embryonic stem cells. Methods Mol Biol 438: 19–30. 18369746 10.1007/978-1-59745-133-8_3

[15] Patani R, Compston A, Puddifoot CA, Wyllie DJ, Hardingham GE, et al (2009) Activin/Nodal inhibition alone accelerates highly efficient neural conversion from human embryonic stem cells and imposes a caudal positional identity. PLoS One 4: e7327. 19806200 10.1371/journal.pone.0007327 PMC2752165

[16] Shi Y, Kirwan P, Smith J, Robinson HP, Livesey FJ (2012) Human cerebral cortex development from pluripotent stem cells to functional excitatory synapses. Nat Neurosci 15: 477–486, S471. 10.1038/nn.3041 PMC3882590 22306606

[17] Chambers SM, Fasano CA, Papapetrou EP, Tomishima M, Sadelain M, et al (2009) Highly efficient neural conversion of human ES and iPS cells by dual inhibition of SMAD signaling. Nat Biotechnol 27: 275–280. 19252484 10.1038/nbt.1529 PMC2756723

[18] Pera MF, Trounson AO (2004) Human embryonic stem cells: prospects for development. Development 131: 5515–5525. 15509763 10.1242/dev.01451

[19] Davila JC, Cezar GG, Thiede M, Strom S, Miki T, et al (2004) Use and application of stem cells in toxicology. Toxicological Sciences 79: 214–223. 15014205 10.1093/toxsci/kfh100

[20] Rolletschek A, Blyszczuk P, Wobus AM (2004) Embryonic stem cell-derived cardiac, neuronal and pancreatic cells as model systems to study toxicological effects. Toxicology Letters 149: 361–369. 15093282 10.1016/j.toxlet.2003.12.064

[21] Pruksananonda K, Rungsiwiwut R, Numchaisrika P, Ahnonkitpanit V, Isarasena N, et al (2012) Eighteen-Year Cryopreservation Does Not Negatively Affect the Pluripotency of Human Embryos: Evidence from Embryonic Stem Cell Derivation. Biores Open Access 1: 166–173. 23514952 10.1089/biores.2012.0242 PMC3559204

[22] Chayosumrit M, Tuch B, Sidhu K (2010) Alginate microcapsule for propagation and directed differentiation of hESCs to definitive endoderm. Biomaterials 31: 505–514. 19833385 10.1016/j.biomaterials.2009.09.071

[23] Huangfu D, Osafune K, Maehr R, Guo W, Eijkelenboom A, et al (2008) Induction of pluripotent stem cells from primary human fibroblasts with only Oct4 and Sox2. Nature Biotechnology 26: 1269–1275. 10.1038/nbt.1502 18849973

[24] Shi Y, Kirwan P, Livesey FJ (2012) Directed differentiation of human pluripotent stem cells to cerebral cortex neurons and neural networks. Nat Protoc 7: 1836–1846. 22976355 10.1038/nprot.2012.116

[25] Huangfu D, Maehr R, Guo W, Eijkelenboom A, Snitow M, et al (2008) Induction of pluripotent stem cells by defined factors is greatly improved by small-molecule compounds. Nature Biotechnology 26: 795–797. 10.1038/nbt1418 PMC6334647 18568017

[26] Claassen DA, Desler MM, Rizzino A (2009) ROCK inhibition enhances the recovery and growth of cryopreserved human embryonic stem cells and human induced pluripotent stem cells. Molecular Reproduction and Development 76: 722–732. 19235204 10.1002/mrd.21021 PMC3257892

[27] Li X, Krawetz R, Liu S, Meng G, Rancourt DE (2009) ROCK inhibitor improves survival of cryopreserved serum/feeder-free single human embryonic stem cells. Human Reproduction 24: 580–589. 19056770 10.1093/humrep/den404

[28] Li X, Meng G, Krawetz R, Liu S, Rancourt DE (2008) The ROCK inhibitor Y-27632 enhances the survival rate of human embryonic stem cells following cryopreservation. Stem Cells and Development 17: 1079–1085. 19006455 10.1089/scd.2007.0247

[29] Watanabe K, Ueno M, Kamiya D, Nishiyama A, Matsumura M, et al (2007) A ROCK inhibitor permits survival of dissociated human embryonic stem cells. Nat Biotech 25: 681–686. 10.1038/nbt1310 17529971

[30] Yao S, Chen S, Clark J, Hao E, Beattie GM, et al (2006) Long-term self-renewal and directed differentiation of human embryonic stem cells in chemically defined conditions. Proc Natl Acad Sci U S A 103: 6907–6912. 16632596 10.1073/pnas.0602280103 PMC1458992

[31] Itsykson P, Ilouz N, Turetsky T, Goldstein RS, Pera MF, et al (2005) Derivation of neural precursors from human embryonic stem cells in the presence of noggin. Mol Cell Neurosci 30: 24–36. 16081300 10.1016/j.mcn.2005.05.004

[32] ten Dijke P, Hill CS (2004) New insights into TGF-beta-Smad signalling. Trends Biochem Sci 29: 265–273. 15130563 10.1016/j.tibs.2004.03.008

[33] Matulka K, Lin HH, Hribkova H, Uwanogho D, Dvorak P, et al (2013) PTP1B is an effector of activin signaling and regulates neural specification of embryonic stem cells. Cell Stem Cell 13: 706–719. 24139759 10.1016/j.stem.2013.09.016

[34] Kirkeby A, Grealish S, Wolf DA, Nelander J, Wood J, et al (2012) Generation of regionally specified neural progenitors and functional neurons from human embryonic stem cells under defined conditions. Cell Rep 1: 703–714. 22813745 10.1016/j.celrep.2012.04.009

[35] Kondo T, Asai M, Tsukita K, Kutoku Y, Ohsawa Y, et al (2013) Modeling Alzheimer’s disease with iPSCs reveals stress phenotypes associated with intracellular Abeta and differential drug responsiveness. Cell Stem Cell 12: 487–496. 23434393 10.1016/j.stem.2013.01.009

[36] Ryan SD, Dolatabadi N, Chan SF, Zhang X, Akhtar MW, et al (2013) Isogenic human iPSC Parkinson’s model shows nitrosative stress-induced dysfunction in MEF2-PGC1alpha transcription. Cell 155: 1351–1364. 24290359 10.1016/j.cell.2013.11.009 PMC4028128

[37] An MC, Zhang N, Scott G, Montoro D, Wittkop T, et al (2012) Genetic correction of Huntington’s disease phenotypes in induced pluripotent stem cells. Cell Stem Cell 11: 253–263. 22748967 10.1016/j.stem.2012.04.026 PMC3608272

