# Supplementary figures and images for: Dual Small-Molecule Targeting of SMAD Signaling Stimulates Human Induced Pluripotent Stem Cells toward Neural Lineages
Source: PLoS One. 2014 Sep 10;9(9):e106952. doi: 10.1371/journal.pone.0106952 (PMC4160199; doi:10.1371/journal.pone.0106952)

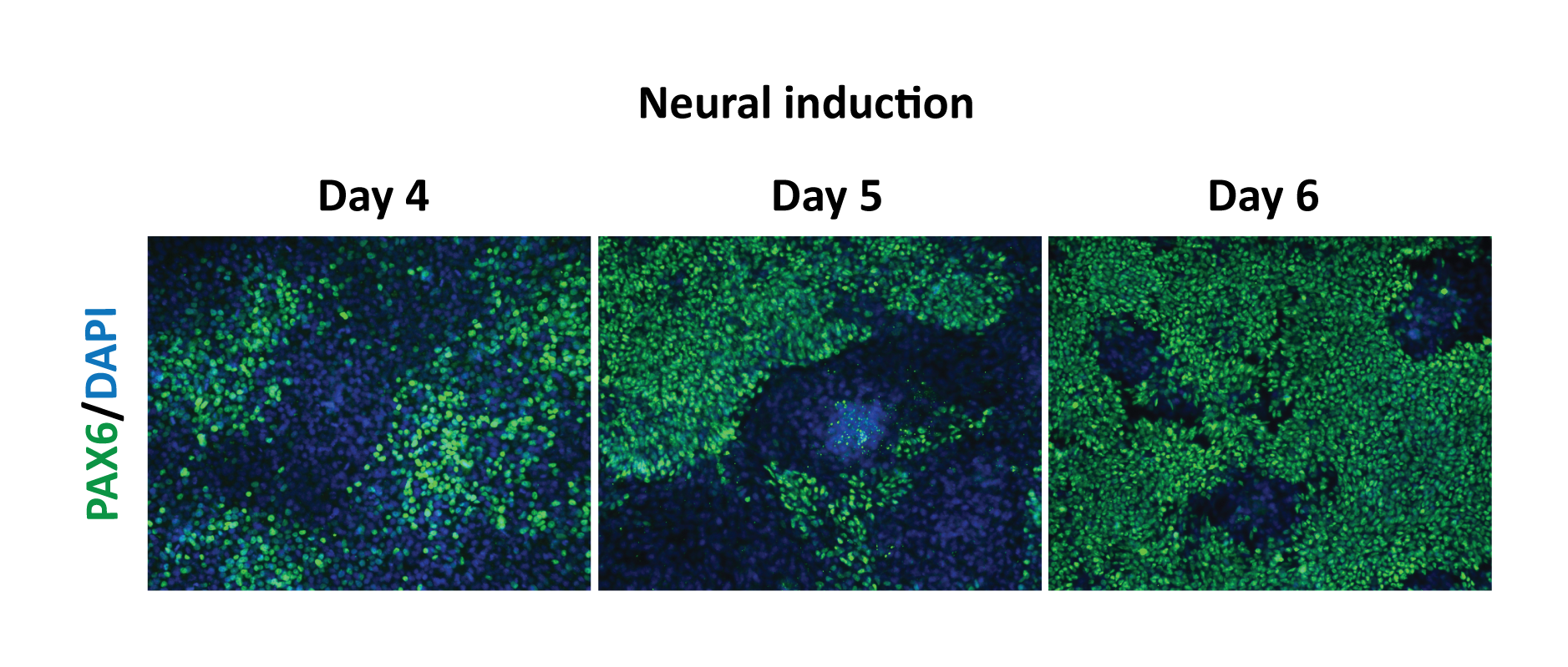

Supplement: Figure S1 — Representative immunofluorescent images show PAX6-positive cells on days 4, 5 and 6 of neural induction. (TIF) [file pone.0106952.s001.tif]

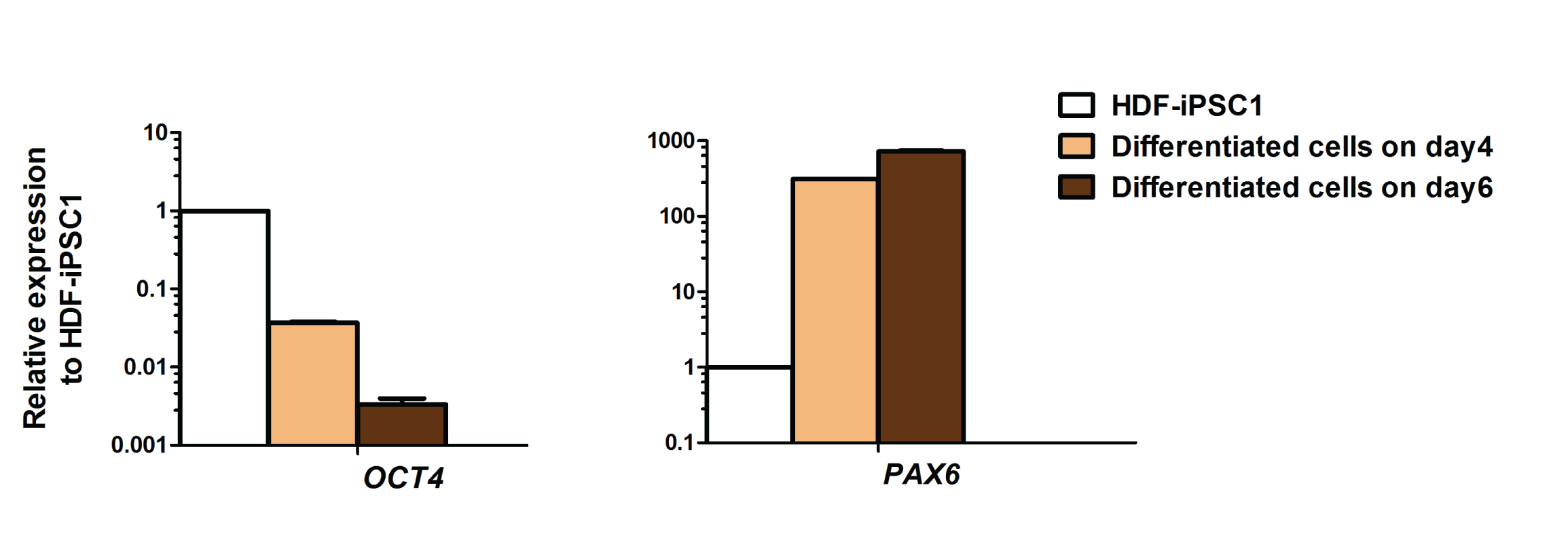

Supplement: Figure S2 — RT-qPCR analysis of OCT4 and PAX6 of differentiated cells on days 4 and 6 of neural induction as compared to those of undifferentiated HDF-iPSC1 cells. (TIF) [file pone.0106952.s002.tif]
