# Supplementary material for: Dual Small-Molecule Targeting of SMAD Signaling Stimulates Human Induced Pluripotent Stem Cells toward Neural Lineages
Source: PLoS One. 2014 Sep 10;9(9):e106952. doi: 10.1371/journal.pone.0106952 (PMC4160199; doi:10.1371/journal.pone.0106952)
Supplement: Table S1 — Primer sequences for RT-qPCR. (DOCX) [file pone.0106952.s003.docx]

**Supplementary Table S1. List of quantitative PCR primers**

| Genes |  | Primer sequence (5’-3’) |
| --- | --- | --- |
| *GAPDH* | Fwd | GTCAACGGATTTGGTCGTATTG |
|  | Rev | CATGGGTGGAATCATATTGGAA |
| *NANOG* | Fwd | AGAAGGCCTCAGCACCTAC |
|  | Rev | GGCCTGATTGTTCCAGGATT |
| *OCT4* | Fwd | TGGGCTCGAGAAGGATGTG |
|  | Rev | GCATAGTCGCTGCTTGATCG |
| *SOX2* | Fwd | AACCCCAAGATGCACAACTC |
|  | Rev | GCTTAGCCTCGTCGATGAAC |
| *C-MYC* | Fwd | TTTCGGGTAGTGGAAAACCA |
|  | Rev | CACCGAGTCGTAGTCGAGGT |
| *KLF4* | Fwd | AGAGTTCCCATCTCAAGGCA |
|  | Rev | GTCAGTTCATCTGAGCGGG |
| *GDF3* | Fwd | TGCTACGTAAAGGAGCTGGG |
|  | Rev | CAGGAGGAAGCTTGGGAAAT |
| *REX1* | Fwd | TCGCTGAGCTGAAACAAATG |
|  | Rev | CCCTTCTTGAAGGTTTACAC |
| *hTERT* | Fwd | TGTGCACCAACATCTACAAG |
|  | Rev | GCGTTCTTGGCTTTCAGGAT |
| *HOXA9* | Fwd | CAATAACCCAGCAGCCAACT |
|  | Rev | CAGTTCCAGGGTCTGGTGTT |
| *ZFPM2* | Fwd | TCATCACATGCAGCAGAACA |
|  | Rev | ATCTCCTGATTGCCCAGATG |
| *SOX1* | Fwd | TCAAGGAAACACAATCGCTG |
|  | Rev | ATTATTTTGCCCGTTTTCCC |
| *FLK1* | Fwd | ACTTTGGAAGACAGAACCAAATTATCTC |
|  | Rev | TGGGCACCATTCCACCA |
| *FOXA2* | Fwd | GGGAGCGGTGAAGATGGA |
|  | Rev | TCATGTTGCTCACGGAGGAGTA |
| *AFP* | Fwd | TGCAGCCAAAGTGAAGAGGGAA |
|  | Rev | ATAGCGAGCAGCCCAAAGAAGA |
| *PAX6* | Fwd | TGGTATTCTCTCCCCCTCCT |
|  | Rev | TAAGGATGTTGAACGGGCAG |
| *OTX1* | Fwd | GCCTCCCCTTCCAGTCTTTC |
|  | Rev | GGGCAGAAACACGCCAGTTA |
| *TUJ1* | Fwd | CCTGGAACCCGGAACCAT |
|  | Rev | AGGCCTGAAGAGATGTCCAAAG |
| *MAP2* | Fwd | CCGTGTGGACCATGGGGCTG |
|  | Rev | GTCGTCGGGGTGATGCCACG |
